# Primary sex determination in birds depends on DMRT1 dosage, but gonadal sex does not determine adult secondary sex characteristics

**DOI:** 10.1073/pnas.2020909118

**Published:** 2021-03-03

**Authors:** Jason Ioannidis, Gunes Taylor, Debiao Zhao, Long Liu, Alewo Idoko-Akoh, Daoqing Gong, Robin Lovell-Badge, Silvana Guioli, Mike J. McGrew, Michael Clinton

**Affiliations:** ^a^Division of Functional Genomics and Development, The Roslin Institute, Royal (Dick) School of Veterinary Studies, EH25 9RG Midlothian, United Kingdom;; ^b^Laboratory of Stem Cell Biology and Developmental Genetics, The Francis Crick Institute, NW1 1AT London, United Kingdom;; ^c^College of Animal Science and Technology, Yangzhou University, 225009 Yangzhou, People's Republic of China

**Keywords:** chicken embryo, gonadal development, testis differentiation, ovary differentiation, sex determination

## Abstract

In birds, males are the homogametic sex (ZZ) and females the heterogametic sex (ZW). Primary sex determination is thought to depend on a sex chromosome gene dosage mechanism, and the most likely sex determinant is the Z chromosome gene Doublesex and Mab-3–Related Transcription factor 1 (DMRT1). To clarify this issue, we used a CRISPR-Cas9–based monoallelic targeting approach and sterile surrogate hosts to generate birds with targeted mutations in the DMRT1 gene. The resulting chromosomally male (ZZ) chicken with a single functional copy of DMRT1 developed ovaries in place of testes, demonstrating the avian sex-determining mechanism is based on DMRT1 dosage. These ZZ ovaries expressed typical female markers and showed clear evidence of follicular development. However, these ZZ adult birds with an ovary in place of testes were indistinguishable in appearance to wild-type adult males, supporting the concept of cell-autonomous sex identity (CASI) in birds. In experiments where estrogen synthesis was blocked in control ZW embryos, the resulting gonads developed as testes. In contrast, if estrogen synthesis was blocked in ZW embryos that lacked DMRT1, the gonads invariably adopted an ovarian fate. Our analysis shows that DMRT1 is the key sex determination switch in birds and that it is essential for testis development, but that production of estrogen is also a key factor in primary sex determination in chickens, and that this production is linked to DMRT1 expression.

## Significance

Here, we show that DMRT1 dosage is the key sex determination factor in birds and is essential for testis development. Furthermore, we provide additional evidence that birds, in contrast to mammals, have acquired cell-autonomous sex identity (CASI) and that the sex hormone environment does not significantly influence avian secondary sexual characteristics. This finding highlights an evolutionary divide between mammals and nonmammalian vertebrates. In mammals, the sex chromosomes determine the type of gonad formed, and sex hormones largely define the secondary sexual phenotype. In birds, the sexual phenotype is directly determined by the sex chromosome content of individual cells in different tissues. Our findings help advance our understanding of the evolution of sex determination systems and the nature of sex identity.

Primary sex determination is the process whereby the developing gonad differentiates into either a testis or an ovary. In general, the genetic factors that regulate gonadal sex differentiation in vertebrates are well conserved although the mechanisms that initiate the process, and the hierarchical interactions of the factors involved, can vary considerably between species. Key conserved male differentiation factors include Doublesex and Mab-3–Related Transcription factor 1 (DMRT1) and anti-Müllerian hormone (AMH) although these are utilized in different ways in different species (1). For example, fishes employ a variety of sex-determining genes, including *dmrt1*, Y-linked *DMRT1* (dmrt1y), sexually dimorphic on Y chromosome (*sdy*), Y-linked AMH (*amhy*), and AMH receptor type-2 (*amhr2*). *Dmrt1* homologs and paralogs, such as W-linked *DMRT1* (*dmw*), are also utilized by some amphibians and reptiles, and sometimes under the control of external stimuli (2–5). Although DMRT1 does not drive primary sex determination in mice and humans, it does play a role of maintaining male somatic cell sex identity in adult testes (1). Factors that play key roles in gonadal female sex determination in many vertebrates are Forkhead box L2 (FOXL2) and estrogen signaling (E_2_). For example, in Tilapia, a Foxl2/Dmrt1 balance appears to control sexual differentiation by regulating E_2_ production through aromatase expression (6). While E_2_ is not a primary sex-determining factor in most mammals, it is able to override genetic sex determination (GSD) in marsupial neonates (7). In chickens, blocking E_2_ synthesis in female embryos leads to masculinization of the gonads while the addition of E_2_ to male embryos leads to feminization of the gonads (8–10).

In birds, the male is the homogametic sex (ZZ), and the female is the heterogametic sex (ZW), but, as yet, there is no evidence for an ovary-determining gene located on the female-specific W chromosome (11). It is widely accepted that primary sex determination in birds is likely to depend on a gene dosage mechanism based on a Z chromosome gene(s) (11). The most likely candidate gene is the Z chromosome gene *DMRT1* (12); *DMRT1* expression is restricted to cells of the gonads and the Müllerian ducts, and it is expressed at higher levels in the male than in the female at the time of sex determination (13, 14). Previous manipulation studies have investigated the link between DMRT1 and sex determination (15), showing that a reduction in DMRT1 levels leads to feminization of the genetically male (ZZ) gonad (16) and that overexpression of *DMRT1* leads to masculinization of the genetically female (ZW) gonad (17).

To elucidate the role of DMRT1 dosage in chicken sex determination, we used an efficient CRISPR-Cas9 targeting approach and surrogate germ cell hosts to generate chickens with targeted mutations in *DMRT1* and analyzed the effects on gonadal development. Here, we clearly demonstrate that avian gonadal sex fate is dependent on *DMRT1* dosage and that the mechanism involves moderation of E_2_ production. The presence of DMRT1 is essential for testicular differentiation, but not for the early stages of ovarian differentiation. Our analysis further supports the concept of cell-autonomous sex identity (CASI) (18) as our results show the development of secondary sexual characteristics of nonreproductive tissues in birds is independent of gonadal sex.

## Results

### Generation of *DMRT1*-Mutant Birds Using Surrogate Hosts.

**Fig. 1.**
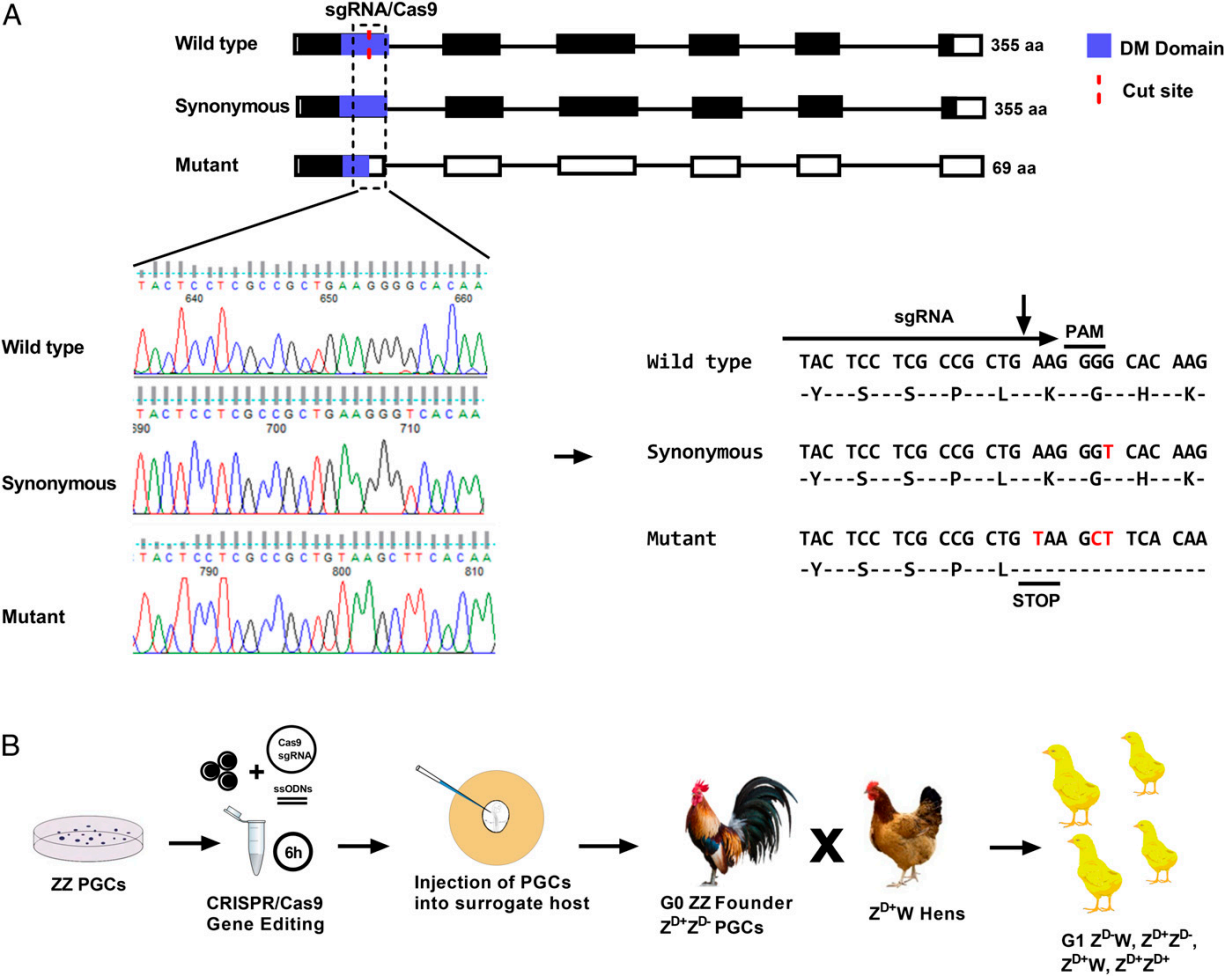
Genome editing of *DMRT1* mutations and genetic crosses. (*A*) Diagram of the *DMRT1* locus in ZZ wild-type and edited ZZ PGC clones carrying a synonymous mutation and a loss of function mutation. The DM DNA binding domain is shown in blue. Details of the Sanger sequencing traces and resulting nucleotide sequences are shown. Nucleotide changes are shown in red. The nonsynonymous change introduced in one allele generates a stop codon and a frameshift in the sequence, resulting in a predicted 69-amino acid (aa) truncated protein, which lacks part of the DNA binding domain. (*B*) Diagram illustrating the overall technical approach and the mating used to produce *DMRT1-*mutant offspring.

To generate *DMRT1* knockout chickens, we used CRISPR-Cas9 to target the *DMRT1* gene in cultured chicken primordial germ cells (PGCs). As DMRT1 is essential for meiosis and gametogenesis in mammals (19, 20), we targeted a loss-of-function mutation into a single *DMRT1* allele in ZZ PGCs (21). ZZ germ cells heterozygous for loss-of-function mutations in essential meiotic genes will successfully navigate meiosis and produce functional gametes (22). We simultaneously delivered a high fidelity CRISPR/Cas9 vector and two single-stranded oligodeoxynucleotides (ssODNs) into in vitro propagated male tdtomato^+^ heterozygote PGCs: one oligonucleotide to create a premature stop codon and a protospacer adjacent motif (PAM) mutation, and a second oligonucleotide, which contained a PAM mutation encoding a synonymous amino acid change in *DMRT1* (*SI Appendix*, Table S1). We isolated clonal male PGC populations and identified clones containing the correct (ZZ *DMRT1*^+/−^; formatted as Z^D+^Z^D-^ for simplicity hereafter) mutations in the *DMRT1* locus (*n* = 10 of 25 clones) (Fig. 1*A*, *SI Appendix*, Fig. S1, and *Methods*).

Targeted (Z^D+^Z^D-^) PGCs were injected into transgenic surrogate host chicken embryos containing an inducible Caspase9 (iCaspase) targeted to the germ cell-specific *DAZL* locus (deleted in azoospermia) (23). Treatment of iCaspase9 host embryos with the dimerization drug AP20187 (B/B) ablates the endogenous germ cells, such that the only gametes that develop are derived from donor PGCs. The surrogate host chicks were hatched and raised to sexual maturity, and then surrogate males (Z^D+^Z^D+^ G_0_ founders carrying Z^D+^Z^D-^ PGCs) were naturally mated to Z^D+^W wild-type hens (Fig. 1*B*). This mating produced chromosomally male and female G_1_ offspring that were wild type for *DMRT1* (Z^D+^Z^D+^ and Z^D+^W), chromosomally male birds that were heterozygous for functional *DMRT1* (Z^D+^Z^D-^), and chromosomally female birds that lacked functional *DMRT1* (Z^D-^W). PCR and red fluorescent protein (RFP) fluorescence expression indicated that 51.6% of DMRT1 embryos were RFP-positive, suggesting that all offspring derived from exogenous PGCs (see *Methods* and *SI Appendix*, Fig. S1*C* and Table S3 for *DMRT1*-allele transmission data).

### ZZ *DMRT1* Heterozygote Embryos Show Gonadal Sex Reversal.

**Fig. 2.**
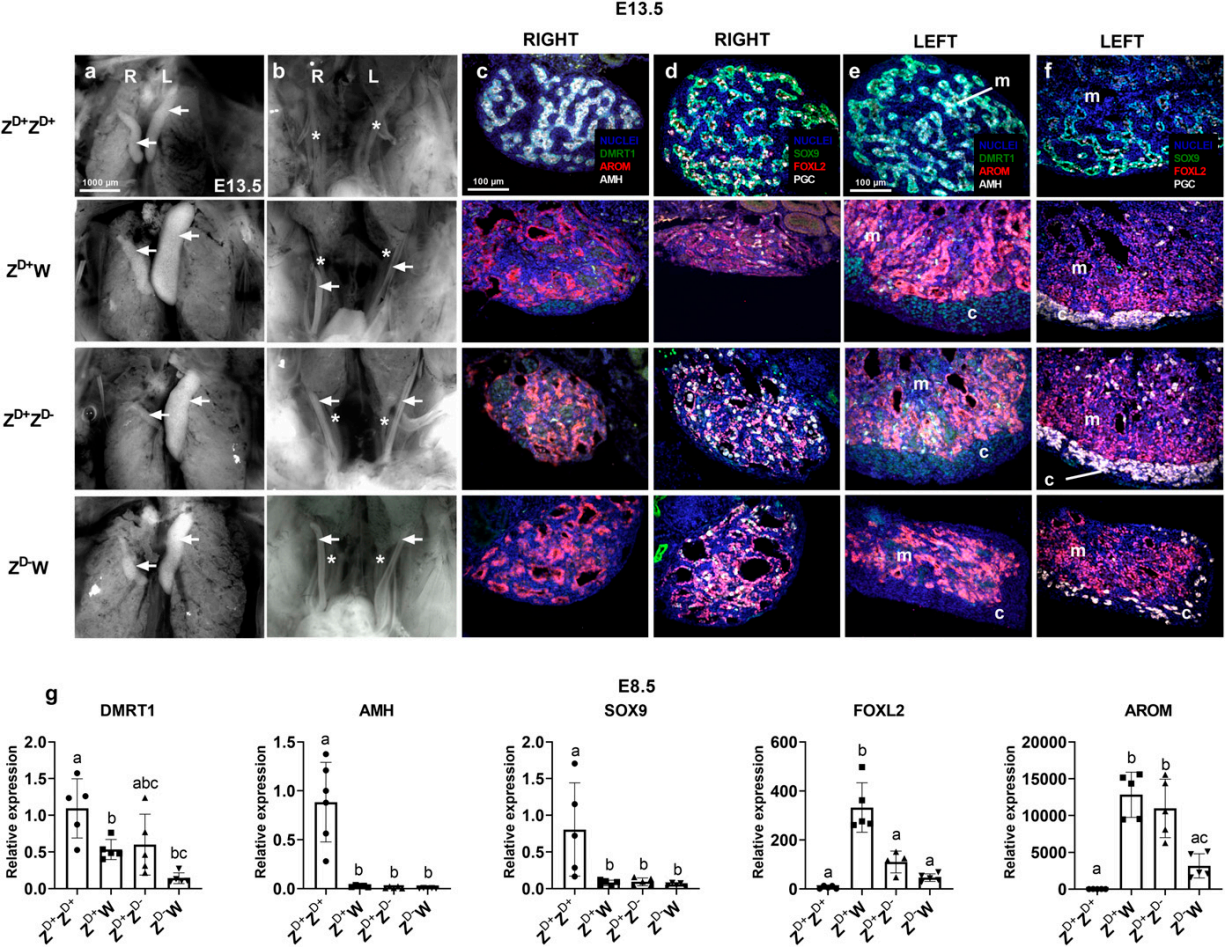
Gonadal development in *DMRT1*-mutant embryos. Gross morphology of gonads (*A*) and Müllerian ducts (*B*) in Z^D+^Z^D+^ and Z^D+^W embryos and Z^D+^Z^D-^and Z^D-^W *DMRT1*-mutant embryos (*n* = 3–7 embryos per genotype). Immuno-sections from right (R) and left (L) gonads from E13.5 wild-type and *DMRT1-*mutant embryos (*C*–*F*). Expression of DMRT1, aromatase (AROM) and AMH (*C*, *E*) and SOX9, FOXL2, and of PGC-specific marker (VASA) (*D*, *F*). A minimum of three embryos of each genotype were examined. Arrows indicate gonads in *A* and Müllerian ducts in *B*. Asterisks indicate Wolffian ducts in *B*. c, cortex; m, medulla. (*G*) Relative gene expression of *DMRT1* and of testis and ovary markers in gonads of E8.5 wild-type and *DMRT1-*mutant embryos. Individual expression levels were calculated relative to levels in Z^D+^Z^D+^. Five replicates on pools of two gonads per genotype. Bars represent mean ± SD. Different letters specify statistically significant groups, *P* < 0.05.

Fertile G_1_ eggs from G_0_ founder males mated to wild-type females were incubated and examined for gonadal development. Our initial characterizations were performed on embryos at day 13.5 of development (E13.5) as clear morphological differences between male and female gonads are apparent by this stage. As expected, in E13.5 ZZ chicken embryos, the testes appeared as two similar sized, cylindrical structures lying on either side of the midline while ZW embryos contained a left ovary, which acquired an elongated flattened appearance, and a small right ovary, which subsequently regressed. The E13.5 testis comprised a core medulla containing germ cell-filled sex cords while the left ovary contained a relatively unstructured medulla surrounded by a thickened cortex containing germ cells (Fig. 2*A*).

Examination of the gross morphology of the gonads in Z^D+^Z^D-^ embryos, however, showed that the targeted mutation of *DMRT1* had a significant effect on gonadal development, with clear morphological signs of sex reversal (Fig. 2*A*). Unlike the typical paired structures seen in the wild-type ZZ embryo, the Z^D+^Z^D-^ clearly contained an ovary-sized structure on the left side and a much smaller structure on the right side, like the Z^D+^W control (*n* = 5 of 5). In Z^D-^W embryos, the left gonad also appeared to be an ovary although smaller in size than the wild-type counterpart (*n* = 3 of 3) (Fig. 2*A*).

It is interesting to note that, by E13.5, both Müllerian ducts had regressed in the Z^D+^Z^D+^ male while both Müllerian ducts were retained in Z^D-^W embryos, similar to Z^D+^W embryos (Fig. 2*B*). This result is unexpected, as it was previously published that down-regulation of DMRT1 blocks Müllerian duct formation (24). We also observed that the right Müllerian ducts of both Z^D-^W and to Z^D+^W embryos showed early signs of regression while, in contrast, the right Müllerian duct of Z^D+^Z^D-^ embryos showed no sign of regression (Fig. 2*B*).

Sections of E13.5 gonads were examined by immunohistochemistry (IHC) to reveal spatial expression patterns of DMRT1 and of established testis (AMH, SRY-box 9 [SOX9]) and ovary (FOXL2, aromatase [Cytochrome P450 Family 19 Subfamily A member 1{ CYP19A1}]) marker proteins, and PGC-specific markers (Fig. 2 *C*–*F*). Sections from both right and left Z^D+^Z^D+^ gonads showed a typical male medulla with obvious sex cords comprised of PGCs and somatic cells that expressed DMRT1, SOX9, and AMH, overlaid by a thin epithelial layer. In contrast, the right and left Z^D+^W gonads were structurally distinct. As expected, the medulla of both right and left gonads expressed FOXL2 and aromatase; however, the right gonad was markedly smaller in size. In addition, the left gonad was enclosed within an obvious thickened cortex on the ventral surface, which contained the PGCs. Analyses of sections of gonads from Z^D+^Z^D-^ embryos revealed that they were indistinguishable from Z^D+^W ovaries in terms of structure and molecular profiles. The medullary regions expressed FOXL2 and aromatase and did not contain sex cords or express SOX9 or AMH. DMRT1 was expressed at low levels, and the left medulla was surrounded by a PGC-containing cortex typical of a Z^D+^W ovary. In Z^D-^W embryos, both gonads were reduced in size, compared to Z^D+^W gonads, but otherwise appeared to be typical ovaries; left and right medullas were FOXL2- and aromatase-positive, and SOX9- and AMH-negative, and the left gonad included a PGC-containing cortex. It is clear from this analysis that the loss of a single functional copy of *DMRT1* leads to ZZ gonadal sex reversal in chickens.

Similar analyses were performed on embryos collected at E5.5, E6.5, and E8.5 (*SI Appendix*, Fig. S2 *A*–*H*). At all stages, the gonads of the Z^D+^Z^D-^ embryos resembled those of wild-type ZW embryos rather than wild-type ZZ embryos and exhibited testis-to-ovary sex reversal. The gonads of Z^D-^W embryos were reduced in size, compared to those of wild-type Z^D+^W embryos at these stages, but otherwise exhibited structural and functional development typical of ovaries. However, we did observe a slight delay in the up-regulation of aromatase in Z^D-^W gonads compared to both Z^D+^Z^D-^ and Z^D+^W embryos (*SI Appendix*, Fig. S2*B*).

To confirm that the introduction of a stop codon into the *DMRT1* locus reduces DMRT1 protein levels in heterozygote and homozygote animals, protein extracts from embryonic stage E8.5 gonads were subjected to a Western blot analysis. We observed a reduction in DMRT1 protein levels in Z^D+^Z^D-^ sex-reversed gonads, compared to Z^D+^Z^D+^ testes, to levels similar to that in Z^D+^W ovaries. A complete loss of DMRT1 protein was observed in Z^D-^W gonads (*SI Appendix*, Fig. S3*A*).

To quantitate the expression of individual gonadal genes, qPCR was performed on RNA extracted from E6.5 and E8.5 gonads. We compared relative expression of *DMRT1* and of testis (*SOX9, AMH*) and ovary (*FOXL2,* aromatase) specific markers in all four genotypes studied. Expression levels at E8.5 relative to expression in Z^D+^Z^D+^ gonads are shown in Fig. 2*G* (E6.5 profiles are shown in *SI Appendix*, Fig. S3*B*). As expected, the expression levels of *DMRT1* in Z^D+^Z^D+^ gonads were approximately twice that seen in Z^D+^W gonads while the levels in the latter and in Z^D+^Z^D-^ gonads were similar. Low levels of mutated *DMRT1* transcripts were detected in gonads of Z^D-^W embryos that purportedly lack full-length DMRT1 protein. Relative to Z^D+^Z^D+^ gonads, expression of the “male” markers *SOX9* and *AMH* was essentially absent in Z^D+^Z^D-^ sex-reversed gonads and equivalent to levels in control Z^D+^W ovaries. In contrast, there was significant expression of the “female” marker *FOXL2* in Z^D+^Z^D-^ gonads. Although *FOXL2* transcript levels in the latter were lower than those in wild-type ovaries, IHC analyses suggested that FOXL2 protein levels were similar (Fig. 2*C*). Expression levels of aromatase in Z^D+^Z^D-^ gonads were similar to those found in control Z^D+^W ovaries. Expression patterns typical of ovaries were also evident in gonads from Z^D-^W embryos completely lacking DMRT1 although the levels of ovary-specific markers were reduced compared to both Z^D+^W and Z^D+^Z^D-^ gonads.

It is clear from these analyses that gonadal development in Z^D+^Z^D-^ embryos is similar to that seen in control ovaries of ZW female embryos.

### Meiosis in *DMRT1*-Mutant Embryos.

**Fig. 3.**
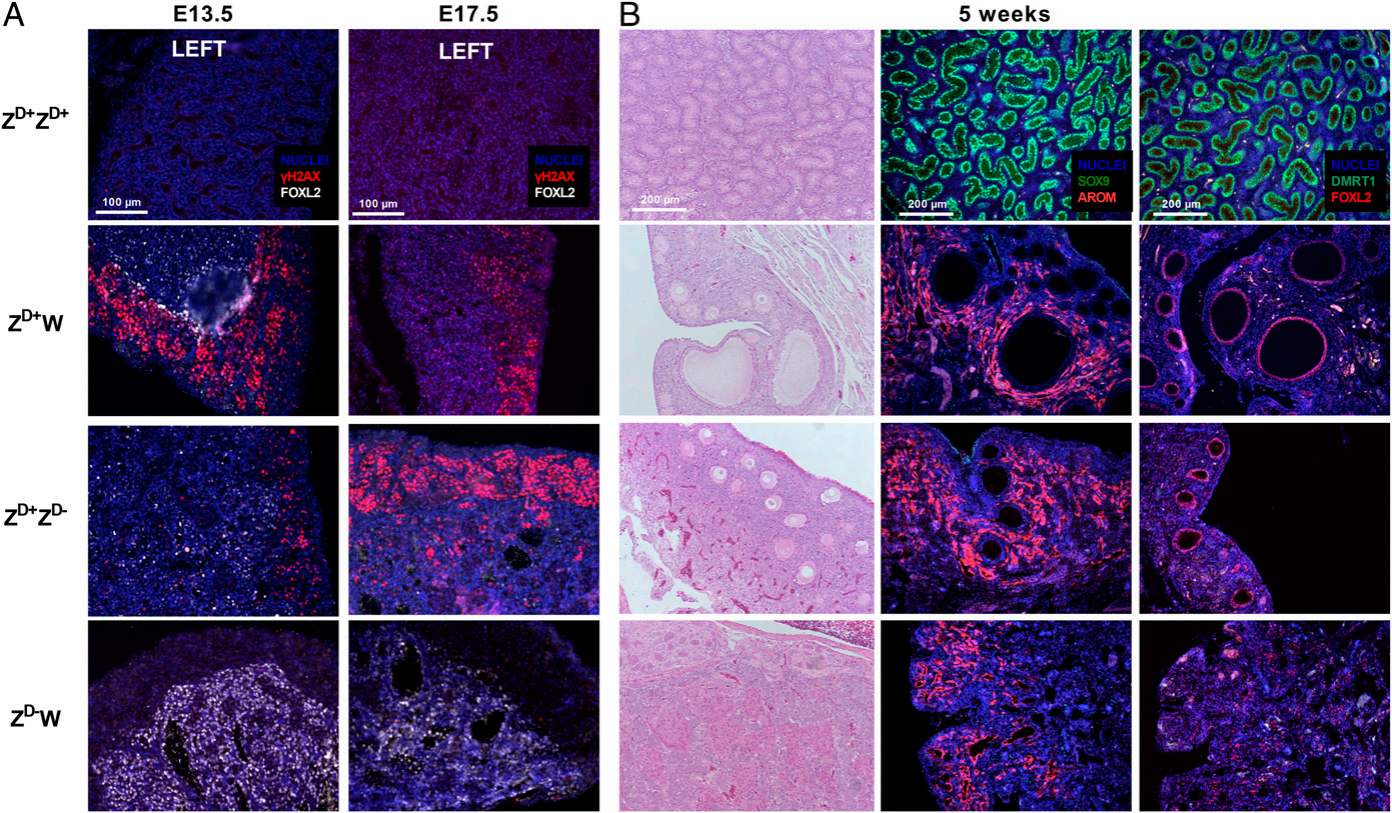
Effect of DMRT1 loss on follicular development. (*A*) FOXL2 and γH2AX expression in germ cells of gonads from wild-type and *DMRT1*-mutant embryos at E13.5 and E17.5 of development (*n* = 3 embryos per genotype). (*B*) Analysis of gonads of wild-type and *DMRT1*-mutant birds at 5 wk posthatch (n = 1–2 per genotype). Sections were stained with either H&E or for testis or ovary-specific markers (FOXL2, AROM, SOX9, and DMRT1).

*DMRT1* is also highly expressed in germ cells and has been implicated in the control of meiotic entry and progression in different vertebrate species (19, 25). To assess the effects of DMRT1 loss on germ cell development, we monitored expression of a selected meiotic marker at E13.5 and E17.5, after the initiation of meiosis in the chicken (Fig. 3*A*). Meiotic progression was assessed by monitoring gamma H2A histone family member X (γH2AX), an indicator of double-stranded DNA breaks (22, 26). As expected, γH2AX was not expressed in germ cells of Z^D+^Z^D+^ gonads at either developmental stage, while germ cells in Z^D+^W gonads expressed γH2AX at both stages with a reduction in expression at E17.5. In the germ cells of gonads from Z^D+^Z^D-^ embryos, γH2AX was present at both stages and, at E17.5, γH2AX expression was more abundant, compared to Z^D+^W controls, indicating a potential delay in meiotic entry in Z^D+^Z^D-^ gonads. In the gonads of Z^D-^W embryos, there was no evidence of γH2AX expression at either developmental stage, suggesting a delay or failure of meiosis.

### Follicular Development in *DMRT1*-Mutant Chicken.

To determine whether the gonadal sex reversal observed during embryonic development was permanent, we examined gonads of birds at 5 wk posthatch. Histological sections of gonads were stained with hematoxylin and eosin (H&E) or processed for IHC to examine expression of male and female markers (Fig. 3*B*). The gonads of Z^D+^Z^D+^ birds exhibited typical testicular structures, with seminiferous tubules showing strong expression of SOX9 and DMRT1. The gonads of Z^D+^W birds displayed a clear cortex with oocyte-containing follicles of different sizes. FOXL2 was highly expressed in the granulosa cells enclosing the oocyte, and aromatase was expressed in the thecal tissue surrounding the follicles. The structure and the expression patterns of FOXL2 and aromatase seen in the gonads of Z^D+^Z^D-^ birds were similar to the Z^D+^W birds, and small follicles were clearly present. However, no larger follicles were observed in Z^D+^Z^D-^ birds. The gonads of Z^D-^W birds contained no oocytes/follicles, and FOXL2 and aromatase were expressed in cells dispersed throughout the cortex. It is clear from this analysis that the testis-to-ovary sex reversal in Z^D+^Z^D-^ birds was permanent and complete. It is well established that DMRT1 is highly expressed in both male and female germ cells, and the absence of oocytes/follicles in the gonads of Z^D-^W birds is likely a direct result of this, leading to an in ovo failure of the germ cells to progress into meiosis. As expected, neither the Z^D+^Z^D-^ nor the Z^D-^W birds produced eggs (*SI Appendix*, Fig. S4*D*).

### Gonadal Sex Reversal Does Not Affect Secondary Sex Characteristics.

**Fig. 4.**
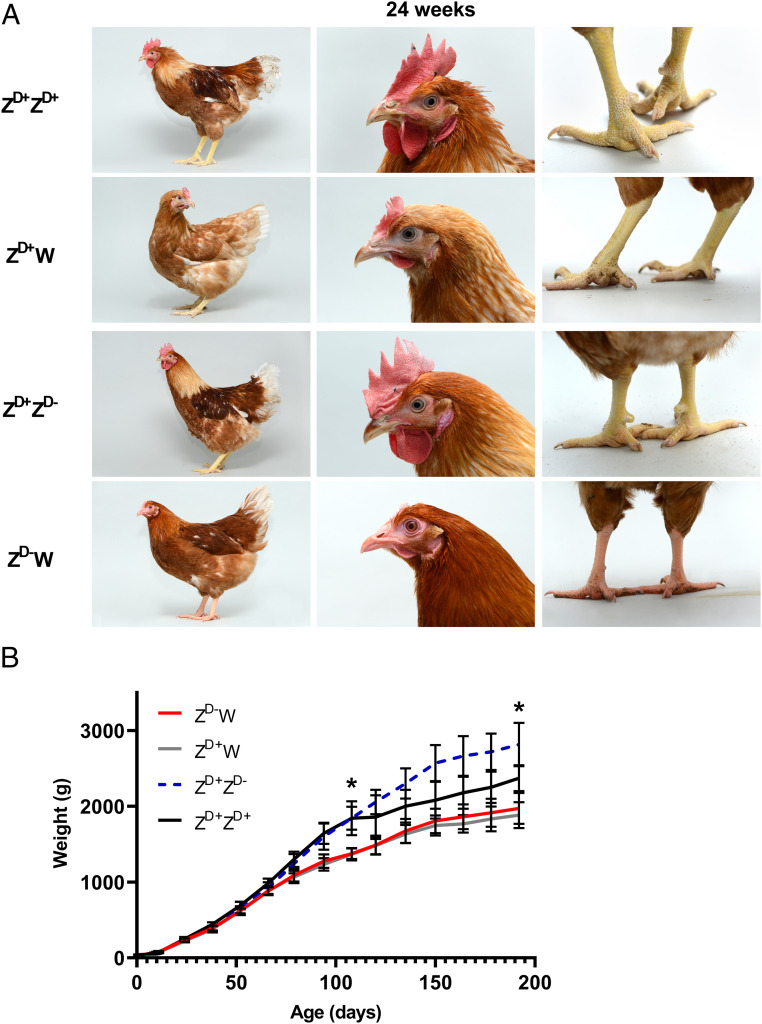
Phenotyping of adult *DMRT1* mutants. (*A*) Physical appearance of wild-type and of *DMRT1*-mutant birds at 24 wk. (*B*) Body weight of wild-type and *DMRT1*-mutant birds. Asterisks indicate a statistically significant difference in body weight between each of the ZZ genotypes (Z^D+^Z^D-^, Z^D+^Z^D+^) and each of the ZW genotypes (Z^D+^W, Z^D-^W), on days 120 and 192 (*n* = 5–8 per genotype). *P* < .05.

We have previously established that chickens possess a degree of CASI: i.e., the secondary sexual phenotype depends, at least partly, on the sex-chromosome content of the somatic cells and not simply on gonadal hormones (18). The generation of Z^D+^Z^D-^ birds that possess an ovary instead of testes enabled us to investigate the extent of CASI in chickens. In terms of secondary characteristics, male birds are heavier (possess greater muscle mass and bone density), they have larger combs and wattles, they possess hackle feathers (hood), and they develop leg spurs (Fig. 4*A*). We assessed sexually mature adult birds at 24 wk of age. It is clear from these images that the chromosomally male bird with an ovary (Z^D+^Z^D-^) was identical in appearance to the wild-type Z^D+^Z^D+^ bird: with large comb and wattles, hackle feathers, and obvious leg spurs. Z^D-^W birds were similar in appearance to Z^D+^W birds. Given that the Z^D+^Z^D-^ bird possesses an ovary rather than testes (*SI Appendix*, Fig. S4*D*), this suggests that these typical male secondary sexual characteristics are due to CASI and independent of gonadal hormones.

We monitored the body weight of wild-type and *DMRT1*-mutant birds over a 28-wk period (Fig. 4*B*). In this line of layer chickens, weights of wild-type male and female birds diverge at 10 wk (70 d), resulting in adult males that were ∼20% heavier than adult females. The Z^D-^W birds followed an almost identical growth pattern to Z^D+^W birds. Z^D+^Z^D-^ birds showed an identical weight increase to Z^D+^Z^D+^ birds up to 120 d but then showed an even greater weight gain until 150 d of age. Postmortem examination suggested that this additional weight accrues from abdominal fat deposits: a phenomenon also associated with capons (27) (castrated cockerels). These results suggest that the weight difference between the Z^D+^Z^D+^ birds and Z^D+^Z^D-^ was due to the loss of testes rather than the acquisition of an ovary. This further suggests that secondary sex characteristics of nonreproductive tissues in chickens are primarily due to the sex chromosome content of cells/tissues and independent of gonadal hormones.

Surprisingly, we observed that the Z^D+^Z^D-^ birds contained mature oviducts derived from both Müllerian ducts; in wild-type male birds, both Müllerian ducts regress while, in wild-type female birds, only the left Müllerian duct is retained, becoming the mature oviduct (*SI Appendix*, Fig. S4 *B* and *C*). In the adult Z^D+^Z^D-^ birds, two mature oviducts were present and connected to the cloaca. Examination of the reproductive ducts of E17.5 embryos showed that, while the right Müllerian ducts of both Z^D+^W and Z^D-^W embryos had fully regressed, the right Müllerian ducts of Z^D+^Z^D-^ embryos exhibited only a slight shortening (*SI Appendix*, Fig. S4*A*). It is well established that wild-type female birds with one oviduct generate low levels of AMH during gonadal development so the retention of both Müllerian ducts in Z^D+^Z^D-^ birds is consistent with a complete loss of AMH expression at embryonic stages (Fig. 2*G* and *SI Appendix*, Fig. S3*B*).

### Female Sex Reversal by E_2_ Blockade Requires DMRT1.

**Fig. 5.**
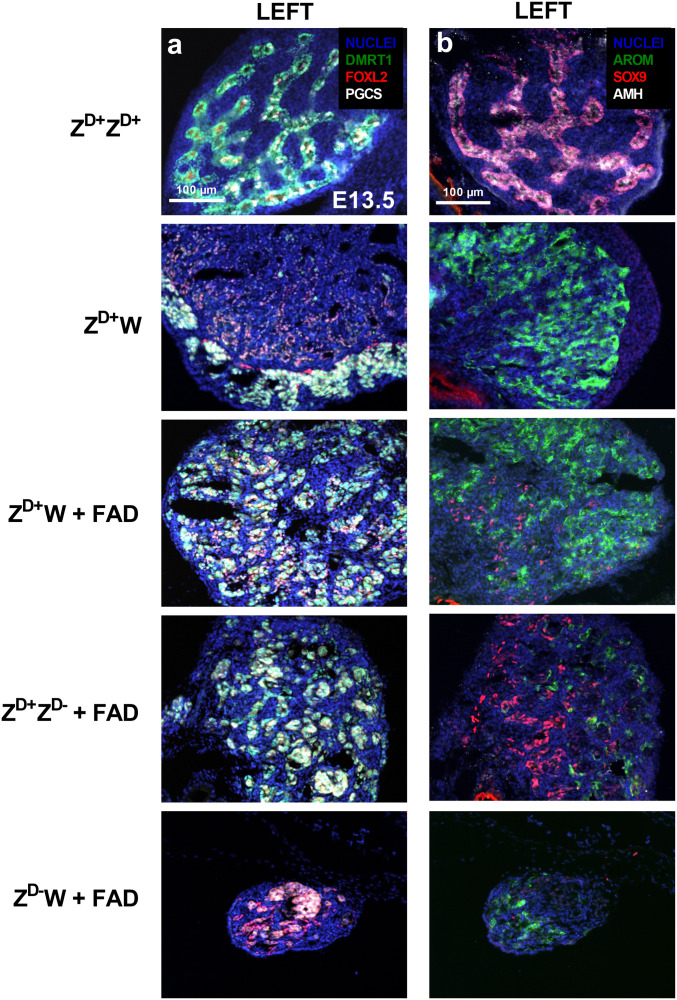
Expression of testis and ovary markers in gonads of fadrozole (FAD)-treated E13.5 embryos. Left gonads are shown. (*A*) IHC of DMRT1, FOXL2, and PGC-marker (VASA). (*B*) IHC of aromatase, SOX9, and AMH. FAD, Fadrozole-treated. Representative of three embryos per genotype.

Multiple reports have established that E_2_ plays a key role in ovarian differentiation in chickens (10, 28). Studies with mixed-sex gonadal chimeras have shown that the presence of a small portion of aromatase-expressing ZW (ovarian) tissue is sufficient to induce cortex formation in the left gonad of wild-type ZZ embryos (10) while it is also well established that blockade of the synthesis of E_2_ in Z^D+^W embryos results in a sex reversal and the gonads develop as testes. Here, we assessed the effects of blocking E_2_ synthesis on gonadal development in *DMRT1* mutants, by injecting E2.5 eggs with an inhibitor of aromatase activity (fadrozole). Following reincubation of eggs, gonads were collected at E13.5 of development and processed for IHC with antibodies against DMRT1 and other gonadal markers (Fig. 5).

The Z^D+^Z^D+^ gonad displayed obvious PGC-containing medullary sex cords with strong DMRT1 and SOX9 expression. The Z^D+^W gonad had a clear PGC-containing outer cortex and displayed medullary expression of FOXL2 and aromatase. The gonads of fadrozole-treated Z^D+^W embryos were clearly affected and showed clear evidence of female-to-male sex reversal; the medulla contained sex cords with germ cells, aromatase expression was reduced and SOX9 expression was evident, and no cortex was present. Z^D+^Z^D-^-treated embryos displayed a similar pattern, demonstrating a rescue of the male-to-female sex reversal phenotype. This indicates that embryos with a single copy of DMRT1 will develop testes in the absence of estrogen. In contrast, fadrozole treatment of Z^D-^W embryos did not result in female-to-male medullary sex reversal: medullary sex cords did not form, and the expression of FOXL2 and aromatase was maintained; however, a thickened cortex is absent.

These findings show that blocking E_2_ synthesis allows testis formation in Z^D+^Z^D-^, but not in Z^D-^W, embryos (Fig. 5). Therefore, although a lack of E_2_ prevents the development of an obvious cortex in fadrozole-treated Z^D-^W embryos, DMRT1 is essential for testis development.

## Discussion

To clarify the role of DMRT1 in sex determination and gonadal development in chickens, we used a CRISPR-Cas9–based approach to generate male offspring carrying disrupting mutations in *DMRT1*. Z^D+^Z^D-^ genome edited PGCs were transmitted through a novel sterile surrogate host, leading to 100% germline transmission. The G_1_ offspring presented the four chromosomal genotypes in a 1:1:1:1 ratio: Z^D+^Z^D+^, Z^D+^W, Z^D+^Z^D-^, and Z^D-^W. The equal transmission of all four possible genotypes demonstrates the Z^D+^ and Z^D-^ spermatozoa formed in the surrogate host gonad (the somatic tissue of which has a Z^D+^ Z^D+^ genotype) were all viable.

The gonads of Z^D+^Z^D-^ embryos resembled the gonads of wild-type female embryos at the equivalent stage at all developmental time points examined (E5.5 to E 17.5). These findings clearly demonstrate that the loss of a single copy of *DMRT1* in male birds results in ovarian rather than testicular development and represent definitive proof of a DMRT1-dependent dosage-based mechanism of sex determination in birds. To determine whether this switch in gonadal fate persisted posthatch, we examined the gonads of these birds at 5 wk of age and again found that these resembled the gonads found in wild-type females. The tissue is clearly ovarian, with a thickened cortex containing follicles, with oocytes surrounded by granulosa and theca layers. Although these ovaries contained significant numbers of small and medium-sized follicles, there was a lack of large follicles, and these birds did not ovulate/lay eggs at sexual maturity. In the wild-type female (Z^D+^W), follicular maturation and ovulation are stimulated by signals from the hypothalamic–pituitary axis (HPA), and the lack of a female HPA in sex-reversed males (Z^D+^Z^D-^) may explain why follicles fail to mature. Alternatively, this failure may be due to subtle defects in Z^D+^Z^D-^ granulosa or theca cells. In any event, the gonads of 5-wk-old Z^D+^Z^D-^ birds are clearly ovarian and demonstrate that the testis-to-ovary sex reversal resulting from the loss of one functional copy of *DMRT1* is a permanent feature.

Unexpectedly, the *DMRT1* Z^D+^Z^D-^ birds were found to contain two mature oviducts. The right oviduct was shorter than the left oviduct, and, in E17.5 embryos, the right Müllerian duct was also shorter than its left counterpart. The mechanism underlying persistence of the right Müllerian duct in Z^D+^Z^D-^ embryos is unclear although regression in Z^D+^W embryos is thought to involve AMH or AMHR2 signaling. In any event, it appears that the retained Müllarian duct tissue is able to respond to the same differentiation signals as the left Müllerian duct and generate a second oviduct. This was surprising as a recent study concluded that DMRT1 was required for the early stages of Müllerian duct development (29). Our findings demonstrate that DMRT1 is not required for Müllerian duct development; the left Müllerian duct forms in Z^D-^W embryos that lack DMRT1 (*SI Appendix*, Fig. S4*D*). It is possible that the different outcomes observed in these studies is due to differences in the timing of DMRT1 depletion. In our study, DMRT1 is absent throughout development whereas, in the earlier study, DMRT1 transcript levels were suppressed in the mesenchyme of the duct during elongation. Perhaps the early depletion of DMRT1 allows for the induction of a factor or factors that compensate for this loss and enable Müllerian duct formation.

We also analyzed gonads of Z^D-^W embryos and found that loss of DMRT1 had little effect on gonadal sex identity, in that female embryos clearly had a left ovary with a thickened cortex containing germ cells. However, when we examined these ovaries at 5 wk posthatch, there were no obvious follicles and no evidence of oocytes although the cortex did contain granulosa cells and theca cells. This suggests that the absence of functional DMRT1 leads to a loss of germ cells in posthatch female birds. Given that *DMRT1* is highly expressed in germ cells and implicated in meiosis in other species, we analyzed meiotic progression in late stage embryos (E13.5 and E17.5) by monitoring the expression of γH2AX, which indicates DNA double-strand breaks typical of meiotic recombination (26). For Z^D+^Z^D-^ embryos, the pattern of marker expression in cortical PGCs was similar, although delayed, to that seen in wild-type female embryos. In contrast, no γH2AX expression was detected in cortical PGCs of chromosomally female embryos lacking DMRT1 (Z^D-^W): a similar PGC phenotype to that observed in *DDX4*-mutant (DEAD-Box Helicase 4) chickens where the germ cells are lost (22). Taken together, these findings suggest that, in these birds, the loss of DMRT1 either prevented or delayed meiosis and resulted in the loss of germ cells.

**Fig. 6.**
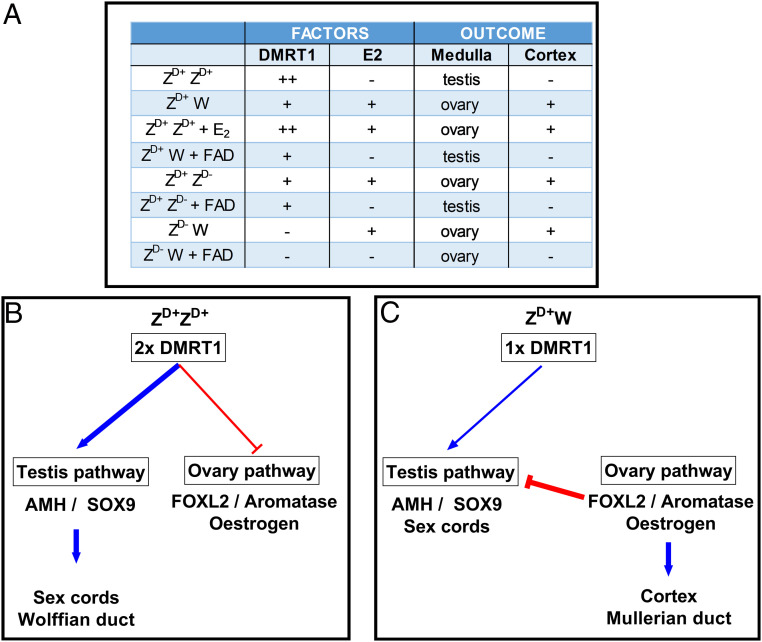
Overview of sex determination in chickens. (*A*) Outcomes resulting from different combinations of DMRT1 and E_2_. (*B* and *C*) Schematics illustrating regulation of gene networks that define male and female reproductive systems (DMRT1: ++/+/− = 2/1/0 copies; E_2_ and Cortex: +/− = present/absent).

It is clear from our studies that the loss of one copy of *DMRT1* in chromosomally male embryos results in the induction of the gene network underlying ovary development: the spatial and temporal expression of first FOXL2 and then aromatase is identical to that seen in wild-type female embryos. This suggests that the presence of two functional copies of *DMRT1* in wild-type male embryos suppresses, either directly or indirectly, the expression of FOXL2. In goats, FOXL2 is a primary ovarian determinant; it has been shown to be a direct activator of aromatase, which catalyzes the conversion of androgens to estrogen (30–32). It is well established that E_2_ also plays a major role in sex determination in birds. Estrogen treatment of chromosomally male embryos leads to ovary formation and inhibition of E_2_ synthesis in chromosomally female embryos results in ovary-to-testes sex reversal (8, 10). In this study, we have investigated the effects of blocking E_2_ synthesis in embryos with targeted mutations in *DMRT1*. We have demonstrated that the left gonad in Z^D+^Z^D-^ embryos develops as an ovary; however, if E_2_ synthesis is blocked in these embryos, both gonads develop as testes. Interestingly, when E_2_ synthesis is blocked in chromosomally female embryos that lack DMRT1, the gonads do not develop as testes, suggesting that DMRT1 is essential for testis formation. The gonad medulla of these embryos continues to express FOXL2 and aromatase, but, because E_2_ synthesis is blocked, cortex formation is not induced. It is noteworthy that the early gonads of Z^D-^W embryos are smaller than those of Z^D+^W embryos, perhaps reflecting a requirement for DMRT1 in the cellular allocation and/or proliferation of the early gonad. Fig. 6*A* summarizes the fate of the gonadal medulla and cortex under the influence of different combinations of DMRT1 and E_2_. We hypothesize that primary sex determination in chickens depends on whether or not the gonadal medulla expresses E_2_. In Z^D+^Z^D+^ embryos, high levels of the Z chromosome DMRT1 suppress FOXL2 expression, which, in turn, leads to a reduction in aromatase expression and to low levels of E_2_ synthesis and allows sex cord formation to be induced. In Z^D+^W embryos, levels of DMRT1 are not sufficient to suppress FOXL2, and the resulting E_2_ inhibits the testis network and induces cortex formation. If E_2_ synthesis is blocked in Z^D+^W embryos, or Z^D+^Z^D-^ embryos, the male pathway is not inhibited, and testis development occurs. If E_2_ synthesis is blocked in embryos devoid of DMRT1 (Z^D-^W), the gonadal medulla resembles that of an ovary, suggesting that DMRT1 is required for testis formation and PGC survival, but it is not necessary for ovary development.

Previously, it was considered that the male and female secondary sexual characteristics of vertebrates were largely dependent on the outcome of primary sex determination and that gonadal hormones played a major role in defining the sexual phenotype. More recently, it has become generally accepted that male:female differences are due to the combined effects of gonadal hormone differences and differences in the sex-chromosome constitution of individual cells and tissues, a classic example being that of marsupial body dimorphism (reviewed in ref. 33). We and others have established that birds possess a CASI and that this plays a major role in defining secondary sexual characteristics (18, 34, 35). Analysis of the adult birds in this study suggest that CASI may be the dominant factor in establishing sexual phenotype and that gonadal hormones have little or no effect on external secondary sexual characteristics. The male birds with ovary in place of testes are virtually identical in growth rate and appearance to wild-type males and display no female characteristics.

Taken together, our findings clearly place DMRT1 dosage in the center of the avian gonadal sex-determining mechanism, while providing evidence for an important role of DMRT1 in germ cell and Müllerian ducts fate. Finally, this work further highlights the unique feature of CASI in birds.

## Methods

### Genome Editing and Generation of *DMRT1* Mutant Birds.

Germ cells were isolated from Hy-line Brown layer embryos heterozygote for an RFP reporter gene (36) at Hamburger–Hamilton (HH) stage 16^+^ and cultured in vitro (37). Briefly, 1 μL of embryonic blood was aspirated from the dorsal aorta of embryos and placed in FAOT (FGF, Activin, ovotransferrin) culture medium (37). Expanded germ cell populations (3 wk) were cotransfected with 1.5 μg of high fidelity CRISPR-Cas9 vector (HF-PX459 V2.0) which included a targeting guide (single guide RNA [sgRNA]) for the *DMRT1* locus and two single-stranded donor oligonucleotides (ssODNs) (5 pmol of each) (*SI Appendix*, Table S1) using Lipofectamine 2000 (Thermo Fisher Scientific) (21). Twenty-four hours after transfection, PGCs were treated with puromycin (at 400 ng/mL) for 48 h to select for edited cells. Following puromycin treatment, PGCs were sorted into single wells of 96-well plates using a FACSAria III (BD Biosciences) at one PGC per well in 110 μL of FAOT to produce clonal populations. PGCs were expanded in culture, DNA was extracted for analysis, and then clonal PGCs were cryopreserved in STEM-CELLBANKER (AMSBIO).

### Generating Surrogate Host Chicken.

Clonal PGCs were thawed, and 1 μL of cells from an individual PGC clone carrying the desired edits for DMRT1 was injected via the dorsal aorta into stage 16 HH^+^ transgenic surrogate host embryos containing an iCaspase9 targeted to the germ cell-specific *DAZL* locus (23, 38). Then, 1.0 μL of 25 mM B/B (in dimethyl sulfoxide [DMSO]) (AP20187; Takara) was added to 50 μL of PGCs (3,000 PGCs per microliter) before injection, and, subsequently, 100 μL of Penicillin/Streptomycin containing 3 μL of 0.5 mM B/B drug (in EtOH) was pipetted on top of the embryo. Treatment of the transgenic surrogate hosts with B/B drug ablates the endogenous germ cells, such that the only gametes that can form are from the donor PGCs. Fourteen surrogate host chicks were hatched from two injection experiments. Four surrogate host chicks carried the iCapsase9 transgene. Two male iCaspase9 surrogate hosts (with somatic genotype Z^D+^Z^D+^), carrying germ cells heterozygous for DMRT1 (Z^D+^Z^D-^), were crossed with wild-type hens (Z^D+^W) to produce G_1_ embryos for analysis and hatched to create G_1_ offspring. All animal experiments were conducted under United Kingdom Home Office license. Experimental protocols and studies were approved by the Roslin Institute Animal Welfare and Ethical Review Board Committee.

### Genetic Screening.

DNA was extracted from cells and embryonic tissues using the PureLink Genomic DNA Mini Kit (Thermo Fisher Scientific) according to the manufacturer’s instructions. To amplify the *DMRT1* locus, PCR reactions included 100 ng of genomic DNA (gDNA) and Q5 high-fidelity polymerase (New England Biolabs) and comprised the following cycling parameters: 98 °C for 2 min, 98 °C for 30 s, 68 °C for 30 s, 72 °C for 30 s, 72 °C for 2 min (steps 2 to 4 run for 32 cycles; Forward primer: CATGCCCGGTGACTCCC; Reverse primer: GAT​CAG​GCT​GCA​CTT​CTT​GC). Gene editing included insertion of an HindIII restriction site, and, to screen clones, PCR products were digested using HF-HindIII (NEB). Enzyme digests were separated by electrophoresis, and genotypes were distinguished by fragment banding patterns (wild-type, monoallelic, and biallelic *DMRT1* mutants) (*SI Appendix*, Fig. S1). All PGC cultures and chicken embryos were sexed using a rapid, invader-based sexing assay (39).

### Tissue Collection.

Freshly laid fertile eggs were incubated blunt side up, at 37.5 °C, in 60% humidity, with rocking (one rotation per 30 min) for the desired incubation period.

Eggs were removed from the incubator at the required stage (E5.5, E6.5, E8.5, E13.5, and E17.5), and embryos were carefully removed and killed according to Home Office Schedule I procedures, and the gonads were dissected and processed for further analysis. Gross morphology of gonads was recorded using a Zeiss Axiozoom Microscope (Carl Zeiss AG).

For RNA analysis, gonads were dissected and placed in phosphate-buffered saline (PBS), and any remaining mesonephric tissue was removed. Gonads were snap-frozen in 10 μL of RNA-Bee (AMS Biotechnology) until RNA extraction. For Western analyses, gonads were collected into 100 μL of radioimmunoprecipitation assay (RIPA) buffer (Thermo Fisher Scientific). For immunostaining, gonads and mesonephroi were placed in 4% paraformaldehyde (see *IHC* section). A small portion of embryonic wing tissue was collected and used to determine genetic sex.

### Quantitative Real-Time PCR.

Individual gonad pairs from E8.5 embryos were homogenized in RNA-bee (AMS Biotechnology), and the lysate was loaded onto a Direct-zol RNA Microprep RNA extraction column (Zymo Research) and DNase-treated as per the manufacturer’s protocol. First-strand complementary DNA (cDNA) was synthesized using the “First-strand cDNA synthesis kit” (GE Healthcare) according to the manufacturer’s instructions. Primers were designed to amplify transcripts from the following genes: *DMRT1, FOXL2, AROM, SOX9, and AMH*. PCR reactions were optimized to meet efficiencies of between 95% and 105% across at least a 100-fold dilution series (primer sequences are listed in *SI Appendix*, Table S1). The qPCR reactions were performed using a Stratagene MX3000P qPCR system (Agilent Technologies). The chicken hydroxymethylbilane synthase gene (HMBS) was used as an internal control (40). Data were analyzed using the 2^-ΔΔCt^ method (41).

### Western Blotting.

Gonads were collected in RIPA buffer (Thermo Fisher Scientific) and disrupted with a handheld homogenizer. Protein levels were quantified using a Pierce BCA protein assay kit (Thermo Fisher Scientific). Protein samples (10 μg) were separated on 4 to 15% Bis-Tris gels (Bio-Rad Laboratories) and wet-transferred onto a polyvinylidene difluoride (PVDF) membrane. Membranes were blocked in Intercept Blocking Buffer for 1 h (LI-COR Biosciences) and incubated overnight with primary antibodies rabbit anti-DMRT1 (42) and rabbit anti–γ-tubulin (T3559; Sigma). After four washes in Tris-buffered saline, 0.1% Tween-20 (TBST), blots were incubated with secondary antibody (horseradish peroxidase [HRP]-conjugated) for 1 h at room temperature, followed by four washes in TBST. Hybridization signals were detected using a Novex chemiluminescence kit (Life Technologies), and membranes were exposed to Hyperfilm ECL (Amersham). Membranes were stripped for 10 min in Restore PLUS Western Blot stripping buffer (Thermo Scientific) for rehybridization.

### IHC.

IHC was carried out according to the protocol described by Stern (43). Gonads were fixed in 4% paraformaldehyde for 2 h at 4 °C. Tissues were equilibrated in 15% sucrose/0.012 M phosphate buffer overnight, embedded in 15% sucrose plus 7.5% gelatin/0.012 M phosphate buffer (pH 7.2), and snap frozen using isopentane. Ten-micrometer-thick sections were cut on a cryostat (OTF 5000; Bright Instruments) and mounted on Superfrost Plus slides (Thermo Fisher Scientific). Slides were degelatinized for 30 min in PBS at 37 °C and blocked in PBS containing 10% donkey serum, 1% bovine serum albumin (BSA), and 0.3% Triton X-100 for 2 h at room temperature. Incubation with primary antibodies (*SI Appendix*, Table S2) was carried out overnight at 4 °C, followed by washing four times in PBS containing 0.3% Triton X-100, and incubation with secondary antibodies for 2 h at room temperature. After washing four times in PBS containing 0.3% Triton X-100, the sections were treated with Hoechst nuclear stain solution (10 μg/mL) for 5 min. Imaging was carried out using a Leica DMLB Upright Fluorescent microscope (Leica Camera AG).

### Data Analysis.

All summary data values are expressed as mean ± SD. GraphPad Prism (Graphpad) was used to produce graphs and for statistical analyses. Statistical analysis of qPCR data included a one-way ANOVA analysis followed by Tukey’s multiple comparison test for post hoc comparisons. *P* < 0.05 was set as the statistical significance threshold.

## Supplementary Material

Supplementary File

## Data Availability

All study data are included in the article and/or *SI Appendix*.
