# Supplementary material for: Primary sex determination in birds depends on DMRT1 dosage, but gonadal sex does not determine adult secondary sex characteristics
Source: Proc Natl Acad Sci U S A. 2021 Mar 3;118(10):e2020909118. doi: 10.1073/pnas.2020909118 (PMC7958228; doi:10.1073/pnas.2020909118)
Supplement: Supplementary File [file pnas.2020909118.sapp.pdf]

SI Appendix

Table S1: Sequences of primers used for RT-qPCR and DMRT1 CRISPR ssODNs.

Target	Forward	Reverse
DMRT1	TTCGCGTTGAGTGCCTCGAC	GAGGACACTGTGAGCCGTTC
FOXL2	ACATGTTCGAGAAGGGCAAC	TGTTCATGAAGGTGGACTGC
SOX9	TACGATTACACCGAGCACCA	GTAGACGGGCTGTTCCCAGT
AMH	TCCACAGCTTTGCCGAGAA	CACATTTTCCCTCTTTGCTCCA
AROM	GCTATTTCCAGCCATTTGGA	TTGGGTGCATGGATAAGTCA
Mutant ssODN	CCCGCTGCCGCAACCACGGCTACTCCTCGGAATTCTAGGTCACAAGCGGTTCTGCTAG TGGCGGGACTGCCAGTGCAAGAAGTGCAGCCTGATC	
Synonymous ssODN	CCCGCTGCCGCAACCACGGCTACTCCTCGCCGCTTAAGGGTCACAAGCGGTTCTGCAT GTGGCGGGACTGCCAGTGCAAGAAGTGCAGCCTGATC	

Table S2: Details of antibodies used for IHC

Target	Details
DMRT1	Rabbit, in-house, 1:500[1]
FOXL2	Goat, Abcam, ab5096, 1:250
SOX9	Rabbit, AbD Serotec, AB5535, 1:250
AMH	Goat, SCBT,SC-6886, 1:250
P450 (AROM)	Mouse, AbD Serotec, MCA20775, 1:250
PGC markers	VASA, Rat, 1:1000[2]<br>DAZL, Abcam, ab215718, 1:500
γH2AX	Mouse, Merck, 05-636, 1:250
γ-tubulin	Mouse ,Sigma, T5326, 1:1000

Table S3: Genotype analysis of *DMRT1*-mutant embryos collected throughout study.

	$Z^{D+}Z^{D+}$	$Z^{D+}W$	$Z^{D+}Z^{D-}$	$Z^{D-}W$	Total
**No. of embryos**	53	43	56	51	203
**Percentage**	26.1%	21.2%	27.6%	25.1%	100.0%

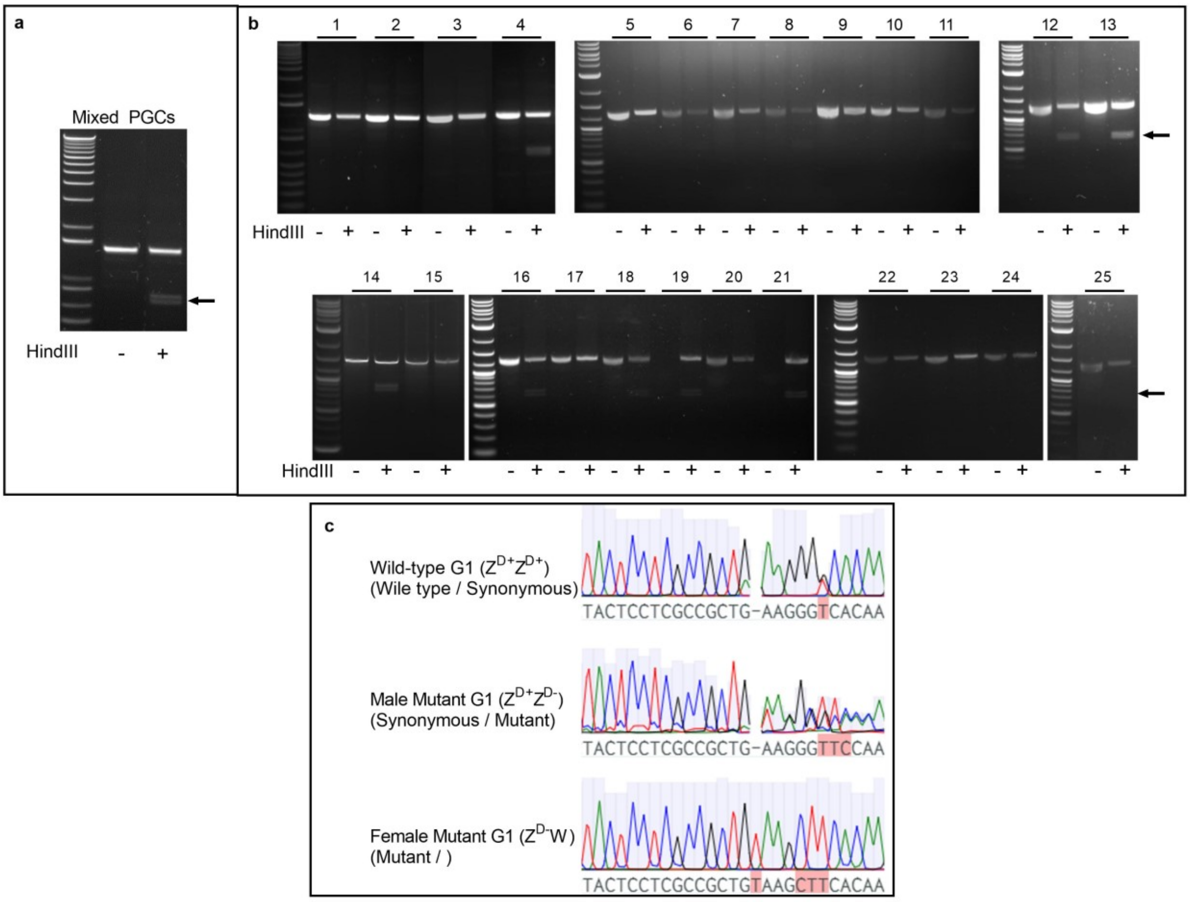

Figure S1: Genotyping analysis of *DMRT1*-edited PGCs and mutants. *Hind*III enzyme was used to analyse the *DMRT1* locus for the 'null' *DMRT1* allele (see Fig 1). Digested *DMRT1* PCR products from puromycin-selected mixed PGCs (a) and clonally cultured PGCs (b). *DMRT1* PCR product: 1.3kb; Restriction fragments: 0.6 kb and 0.7 kb. c) Representative Sanger sequencing chromatograms of the DMRT1 target region in G1 wild-type and mutant embryos, confirming the mono-allelic mutation of DMRT1 in $Z^{D+}Z^{D-}$ mutants.

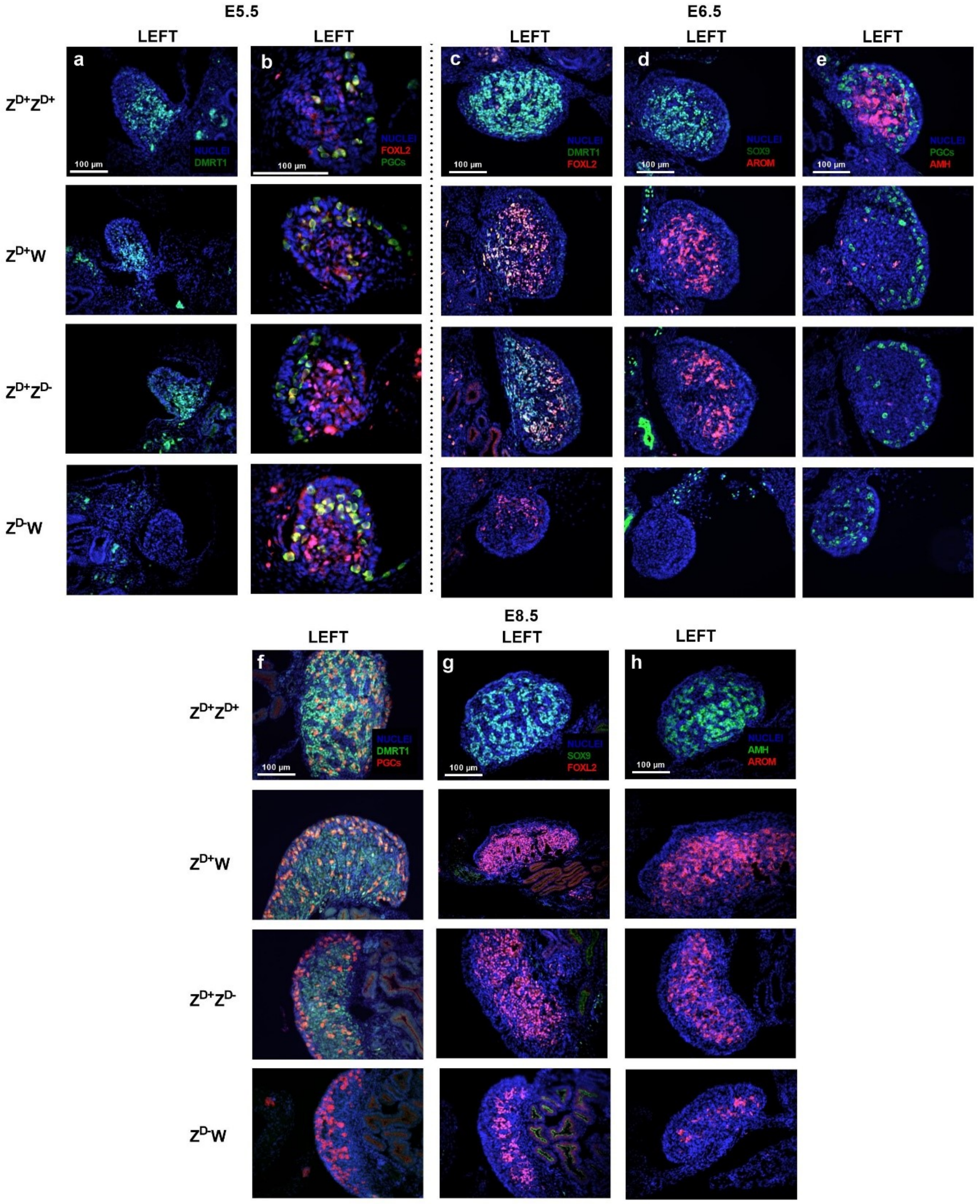

Figure S2: Immunostaining of gonadal sections from E5.5 (a-b), E6.5 (c-e) and E8.5 (f-h) *DMRT1*-mutant embryos. Antibodies were against DMRT1, FOXL2, SOX9, AMH, aromatase (AROM) and germ cell markers (DAZL at E5.5 and E6.5, VASA at E8.5). Aromatase, SOX9 and AMH were not detected in E5.5 sections. There was no staining for aromatase in the $Z^{D-}W$ embryos on E6.5, suggesting a delay in initiating transcription. A minimum of three embryos of each genotype were examined.

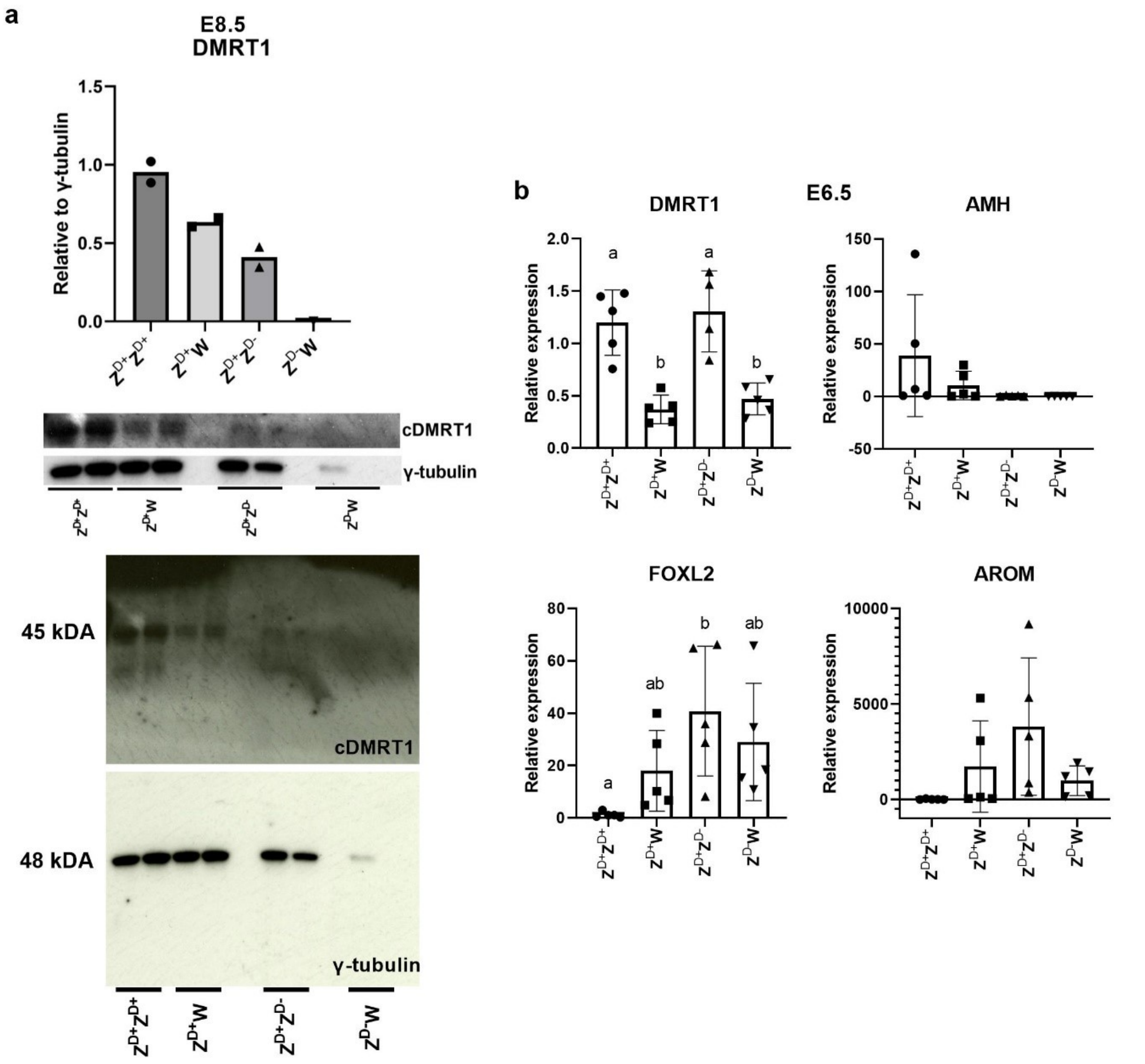

Figure S3: Western blots (a) on *DMRT1*-mutant and wild type control gonads at E8.5, including full membrane images for each antibody as annotated. Histogram bars represent the mean intensity value derived from two lanes per genotype (with the exception of $Z^{D-}W$). RT-qPCR profiling (b) of key gonadal markers in gonads from E6.5 *DMRT1*-mutant embryos (pool of five gonad pairs per data point). The degree of variation in expression seen within each genotype is likely to reflect slight differences in stage of development: *AMH*, *FOXL2* and *Aromatase* (AROM) sex-biased expression initiates around this stage. Note the low expression of *DMRT1* transcript in $Z^{D-}W$ gonads; this is attributed to the *DMRT1* primers detecting mutant *DMRT1* transcript, which is still produced (however, the mutant stop codon prevents translation of wild type protein). Bars represent the mean ± standard deviation relative to $Z^{D+}Z^{D+}$. Different letters signify statistically different expression between individual genotypes (see Methods).

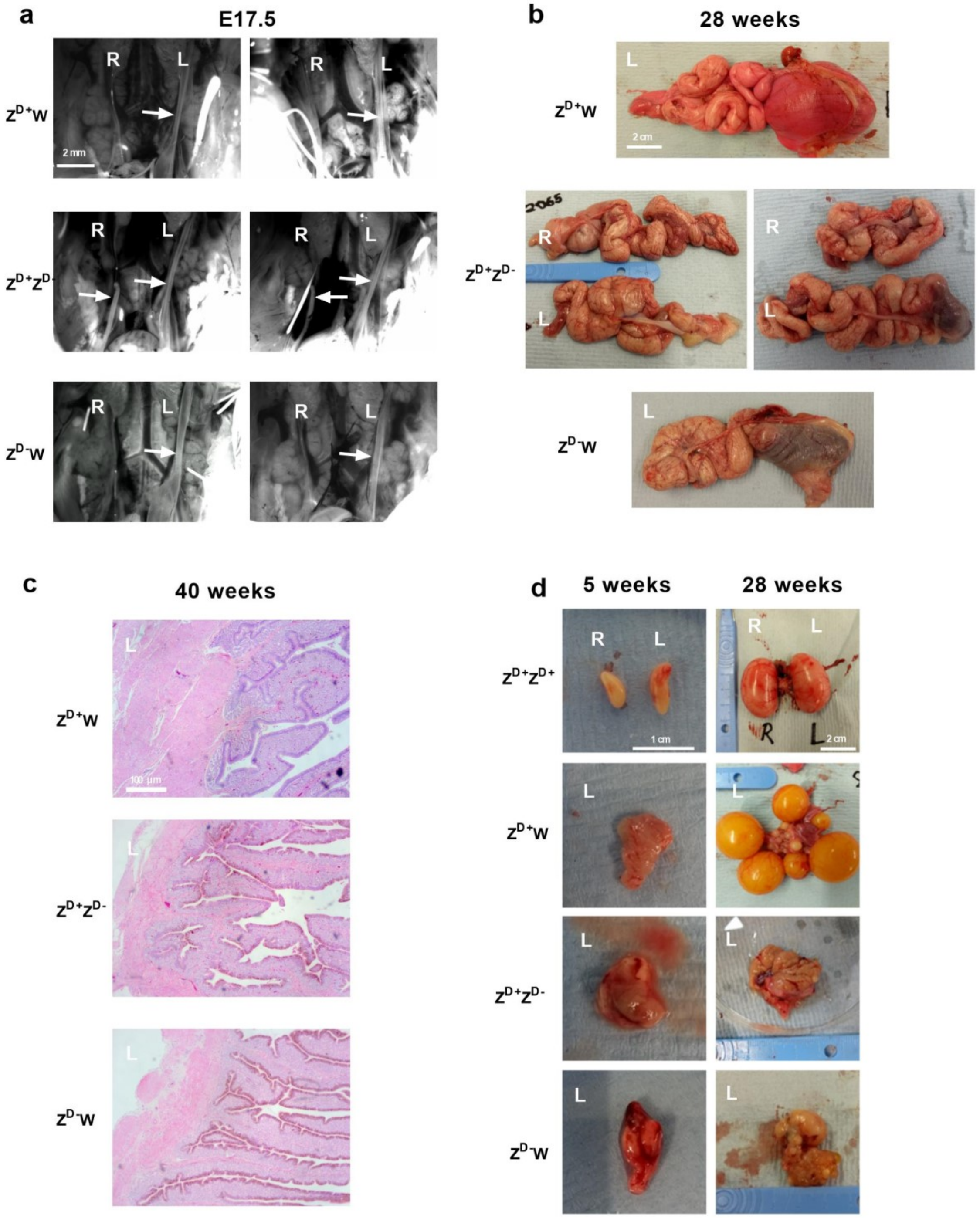

Figure S4: Gross reproductive morphology of Mullerian ducts and gonads of *DMRT1* mutants. Images of Mullerian ducts (a) and oviducts (b) from *DMRT1* mutants and wild type controls at E17.5 and 28 weeks, respectively. c) Histological slides (H&E) of oviducts from *DMRT1* mutants and wild type controls at 40 to 45 weeks. Note the pigmentation in the epithelium of the non-laying genotypes $Z^{D+}Z^{D-}$ and $Z^{D-}W$. No histological abnormalities noted for the oviduct tissues examined. d) Photographs of *DMRT1*-mutant gonads and wild type controls at 5 weeks and 28 weeks post-hatch. Arrows in (a): retained right Mullerian ducts. At least two replicates used per genotype.

SI References

1. Guioli, S. & Lovell-Badge, R. PITX2 controls asymmetric gonadal development in both sexes of the chick and can rescue the degeneration of the right ovary. *Development* **134**, 4199-208 (2007).
2. Aramaki, S., Kubota, K., Soh, T., Yamauchi, N. & Hattori, M.A. Chicken dead end homologue protein is a nucleoprotein of germ cells including primordial germ cells. *J Reprod Dev* **55**, 214-8 (2009).
